# Computational modelling of placental amino acid transfer as an integrated system

**DOI:** 10.1016/j.bbamem.2016.03.028

**Published:** 2016-07

**Authors:** N. Panitchob, K.L. Widdows, I.P. Crocker, E.D. Johnstone, C.P. Please, C.P. Sibley, J.D. Glazier, R.M. Lewis, B.G. Sengers

**Affiliations:** a Bioengineering Science Research Group, Faculty of Engineering and the Environment, University of Southampton, UK; b Maternal & Fetal Health Research Centre, Institute of Human Development, University of Manchester, UK; c St. Mary's Hospital & Central Manchester University Hospitals NHS Foundation Trust, Manchester Academic Health Science Centre, UK; d Faculty of Medicine, University of Southampton, UK; e Mathematical Institute, Oxford University, Oxford, UK; f Institute for Life Sciences, University of Southampton, UK

**Keywords:** Amino acids, Placenta, Epithelial transport, Mathematical model

## Abstract

Placental amino acid transfer is essential for fetal development and its impairment is associated with poor fetal growth. Amino acid transfer is mediated by a broad array of specific plasma membrane transporters with overlapping substrate specificity. However, it is not fully understood how these different transporters work together to mediate net flux across the placenta. Therefore the aim of this study was to develop a new computational model to describe how human placental amino acid transfer functions as an integrated system. Amino acid transfer from mother to fetus requires transport across the two plasma membranes of the placental syncytiotrophoblast, each of which contains a distinct complement of transporter proteins. A compartmental modelling approach was combined with a carrier based modelling framework to represent the kinetics of the individual accumulative, exchange and facilitative classes of transporters on each plasma membrane. The model successfully captured the principal features of transplacental transfer. Modelling results clearly demonstrate how modulating transporter activity and conditions such as phenylketonuria, can increase the transfer of certain groups of amino acids, but that this comes at the cost of decreasing the transfer of others, which has implications for developing clinical treatment options in the placenta and other transporting epithelia.

## Highlights

• First computational model of placental amino acid transfer as an integrated system
• Increased activity of a transporter does not mean increased transfer to the fetus.
• Increasing transfer of certain amino acids can reduce the transfer of others.
• Amino acid composition as well as concentration determines transfer to the fetus.
• Modelling of phenylketonuria suggests inhibition by excess maternal phenylalanine.

## Graphical abstract

**Figure:**
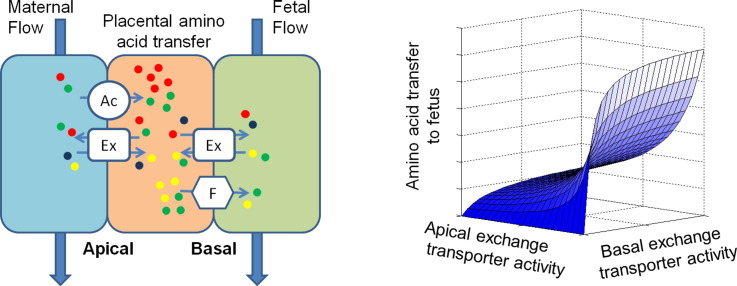

## 1 Introduction

The placenta is the interface between the maternal and fetal circulations and plays an essential role in mediating the transfer of all the nutrients required for fetal development, including amino acids. Impaired placental transfer of amino acids during pregnancy is associated with poor fetal growth, which increases the risk of poor pregnancy outcomes such as stillbirth [1] and of chronic disease in adult life [2], [3], [4]. There are currently no effective treatments for fetal growth restriction (FGR) and a better understanding of placental transfer as a whole could potentially contribute to the development of treatment strategies for intervention and prevention of the disease.

Transfer of amino acids across the placenta is a complex process, influenced by multiple factors including placental blood flow, membrane transporters, intracellular metabolism and placental morphology [5], [6]. In order to pass from the maternal intervillous space into the fetal capillaries, amino acids need to cross the placental syncytiotrophoblast, an epithelial barrier separating the two circulations. Amino acids in the maternal blood first need to be transported across the microvillous plasma membrane (MVM) of the placental syncytiotrophoblast into the cytosol. They can then either undergo metabolism or can be transported across the fetal-facing basal plasma membrane (BM), from where it is assumed they diffuse across the fetal capillary endothelium to the fetal circulation [6].

Amino acid transport across the MVM and BM is mediated by specific transport proteins [6], which operate using different energetically passive and active transport mechanisms. Accumulative transporters actively pump amino acids into the placental syncytiotrophoblast against their concentration gradient, using secondary active transport driven by the sodium electrochemical gradient. This serves as an important driving force for amino acid transfer as a whole, since fetal amino acid concentrations are higher than maternal concentrations [7] and syncytiotrophoblast cytosol concentrations are higher than both [8]. Exchangers (antiporters) are another important class of transporter, which take one amino acid from outside of the plas`ma membrane and swap it for another amino acid from inside the syncytiotrophoblast. Thus, exchangers mediate changes in the relative amino acid composition but not the overall net amount. Facilitative transporters on the other hand are responsible for mediating net transport to the fetus, via facilitative diffusion driven by the amino acid electrochemical gradients [9], [10].

Critically, these three classes of transporter need to work together to mediate net transfer of all the required amino acids to the fetus, as it is not possible for one to do so alone [9], [11]. For example, substrates taken up by the accumulative transporter across the MVM can be exchanged back to the mother to drive uptake by exchangers of amino acids that are not substrates of the accumulative transporter. Similarly, the exchangers at the BM transfer amino acids to the fetus that are not substrates of the facilitative transporters.

While many studies of amino acid transfer have focussed on individual transporters, the integrated study of the interactions between multiple transporters in the two placental plasma membranes has been limited [9], [12]. There are twenty amino acids, which can either inhibit or promote each other's transport, and many distinct transporter proteins with overlapping substrate specificity. Hence, given this inherent complexity, a systems approach using mathematical modelling is necessary to help describe the transport process as a whole. Previous placental models have mainly focussed on blood flow and oxygen transport by simple diffusion, which has proved highly valuable to explain placental structure–function relationships [13], [14], [15], [16], [17], while models for membrane transport have been applied for the placental transfer of drugs [18] and glucose [19]. We have previously introduced a model of human placental amino acid transfer, applied to the uptake and exchange of serine and alanine [20]. However, a systematic integrated analysis of amino acid transfer is required, including more mechanistic transporter models [6], [21], [22].

The aim of this study was to develop a modelling framework for human placental amino acid transfer as an integrated system, to better understand (i) how different types of transporter work together, (ii) how composition of amino acids affects transport, and (iii) how specific transporter activities can drive net transfer of all amino acids to the fetus.

## 2 Methods

### 2.1 Compartmental model for the placenta

**Fig. 1:**
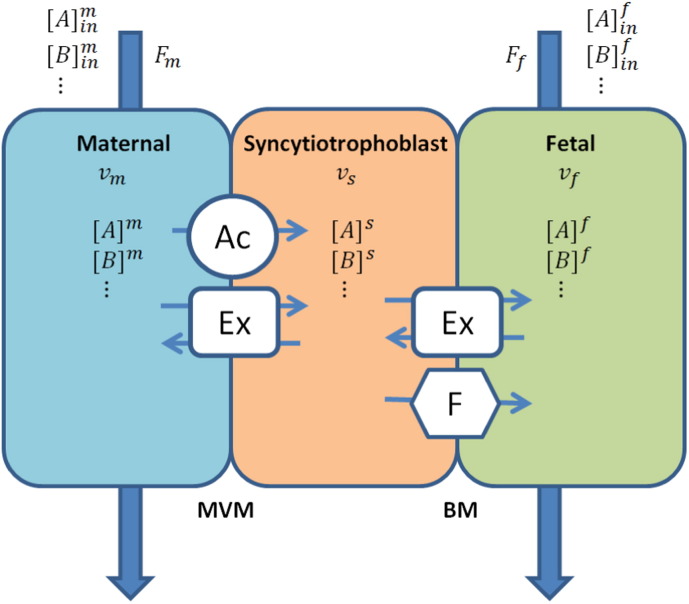
Compartmental model of the placental amino acid transfer system, representing the volumes of the maternal intervillous space (*v*_*m*_), placental syncytiotrophoblast (*v*_*s*_), and fetal capillaries (*v*_*f*_). Amino acid transporters included at the maternal-facing microvillous membrane (MVM) were accumulative transporter (Ac) and exchanger (Ex), and those at the fetal-facing basal membrane (BM) were facilitative transporter (F) and exchanger (Ex). *F*_*m*_ and *F*_*f*_ are the flow inputs (and outputs) in the maternal and fetal compartments respectively. [*A*] and [*B*] indicate amino acid concentrations in the various compartments.

A compartmental modelling approach was adopted based on our previous work [20], in which the placenta was represented as three separate volumes, corresponding to the maternal intervillous space, syncytiotrophoblast, and fetal capillaries respectively (Fig. 1). All compartments were assumed to be well mixed, as the main focus is on the transporter interactions. The transfer of amino acids between compartments was modelled as fluxes mediated by the various types of transporters [9]. In each membrane (MVM and BM), transport by a certain type of transporter was combined and modelled as a single representative transporter. At the maternal-facing MVM these included transport by an accumulative and an exchange transporter, while at the fetal-facing BM transport by a facilitative and an exchange transporter (Fig. 1). Note that accumulative transporters are also found on the BM, but these were not included in the model as their role is thought to be limited [11]. Details of the model implementation are described below.

The rate of change in the concentration of a certain amino acid *A* within each placental compartment is given by:

(1) $$\frac{d{\left[A\right]}^{m}}{\mathrm{dt}}=\frac{1}{v_{m}}\left(J_{A,\mathrm{flow}}^{m}-J_{A,\mathrm{ac}}^{m\to s}-J_{A,\;\mathrm{ex}}^{m\to s}\right)$$

(2) $$\frac{d{\left[A\right]}^{s}}{\mathrm{dt}}=\frac{1}{v_{s}}\left(J_{A,\mathrm{ac}}^{m\to s}+J_{A,\mathrm{ex}}^{m\to s}-J_{A,\mathrm{ex}}^{s\to f}-J_{A,\;\mathrm{fa}}^{s\to f}\right)$$

(3) $$\frac{d{\left[A\right]}^{f}}{\mathrm{dt}}=\frac{1}{v_{f}}\left(J_{A,\mathrm{flow}}^{f}+J_{A,\mathrm{ex}}^{s\to f}+J_{A,\mathrm{fa}}^{s\to f}\right)$$

where *[A]*^*i*^ is the concentration (mol l^− 1^) of substrate *A* in compartment *i*, and *v*_*i*_ is the compartment volume (l). *J*_*A*_^*i* → *j*^ represent the net molecular flux (mol min^− 1^) of *A* from compartment *i* to *j.* Here *m*, *s*, and *f*, are the maternal, syncytiotrophoblast and fetal compartments respectively, while *ac*, *ex*, and *fa* denote the accumulative, exchange, and facilitative transporters. *J*_*A* , *flow*_^*i*^ is the net molecular flux (mol min^− 1^) due to blood flow.

### 2.2 Classification of amino acids in representative groups

Amino acids were categorised according to their transporter specificity into four generic groups, to reduce complexity in the first instance. As shown in Table 1, these representative amino acid groups were AcEx, substrate of the accumulative and exchange transporters; Ex, exchange only substrate; ExF, substrate of exchange and facilitative transporters; and AcExF, substrate of all transporter types. Normal physiological concentrations of amino acids [7], [8] were summed per representative group in each compartment. Glutamate and aspartate were not included in the model as these are taken up by distinct transport systems (EAATs) and their interactions with the transfer of other amino acids would be extremely limited.

### 2.3 Individual transporter models

Models for each type of transporter were developed based on the principles of carrier-mediated transport [21], [23], to represent the simultaneous transport of multiple substrates. Briefly, parameters describing the kinetic properties were kept to the minimum required to represent the functional activity of each transporter type. Therefore, in the first instance, exchanger and facilitative transporter translocation rate constants were assumed to be symmetric and binding affinities equal on both sides of the plasma membrane. In addition, substrates of a certain type of transporter were all assumed to have identical kinetic and binding properties.

#### 2.3.1 Exchanger model

For the exchanger with multiple substrates, the net flux of substrate *A* from compartment *I* to *II* (mol min^− 1^) is given by [24]:

(4) $$J_{A,\mathrm{ex}}^{I\to \mathrm{II}}=V_{\mathrm{ex}}\frac{{\left[A\right]}^{I}{\left[R\right]}^{\mathrm{II}}-{\;\left[A\right]}^{\mathrm{II}}{\left[R\right]}^{I}}{K_{\mathrm{ex}}\left({\left[\mathrm{Tot}\right]}^{I}+{\left[\mathrm{Tot}\right]}^{\mathrm{II}}\right)/2+{\left[\mathrm{Tot}\right]}^{I}{\left[\mathrm{Tot}\right]}^{\mathrm{II}}}$$

where *[A]*^*i*^ is the concentration of substrate *A* (mol l^− 1^) in compartment *I* or *II*, *[Tot]*^*i*^ is the total sum of all exchanger substrates, while *[R]*^*i*^ denotes the sum of all exchanger substrates, but excluding substrate *A* in compartment *i*. *K*_*ex*_ is the dissociation constant (mol l^− 1^), assumed equal for all substrates of the exchanger, and *V*_*ex*_ is the maximum transport rate (mol min^− 1^).

#### 2.3.2 Facilitative transporter model

For the facilitative transporter with multiple substrates, the net flux of substrate *A* from compartment *I* to *II* (mol min^− 1^) is given by [24]:

(5) $$J_{A,\mathrm{fa}}^{I\to \mathrm{II}}=V_{\mathrm{fa}}\left(\frac{{\left[A\right]}^{I}}{K_{\mathrm{fa}}+{\left[\mathrm{Tot}\right]}^{I}}-\frac{{\left[A\right]}^{\mathrm{II}}}{K_{\mathrm{fa}}+{\left[\mathrm{Tot}\right]}^{\mathrm{II}}}\right)$$

where *[A]*^*i*^ is concentration of substrate *A* (mol l^− 1^) in compartment *i*, *[Tot]*^*i*^ is the sum of the concentrations of all substrates of the facilitative transporter in compartment *i*. *K*_*fa*_ is the dissociation constant (mol l^− 1^), assumed equal for all substrates of the facilitative transporter, and *V*_*fa*_ is the maximum transport rate (mol min^− 1^).

#### 2.3.3 Accumulative transporter model

Accumulative transporters operate via secondary active transport driven by the sodium electrochemical gradient. A cotransport model was adopted [24], in which it was assumed that sodium binds to the transporter first, followed by the amino acid [25] and that only the translocation of the carrier-sodium-substrate complex was electrogenic. The net flux of substrate *A* from compartment *I* to *II* (mol min^− 1^) is given below for the model distinguishing two different substrates:

(6) $$\begin{array}{l} J_{A,\mathrm{ac}}^{I\to \mathrm{II}}=\frac{V_{\mathrm{ac}}}{D/2}\left\{\varepsilon \varepsilon \prime {\left[\mathrm{Na}\right]}^{I}{\left[\mathrm{Na}\right]}^{\mathrm{II}}\left({\left[A\right]}^{I}{\left[B\right]}^{\mathrm{II}}-{\left[A\right]}^{\mathrm{II}}{\left[B\right]}^{I}\right)+K_{\mathrm{ac}}K_{\mathrm{Na}}\left(\varepsilon \prime {\left[A\right]}^{I}{\left[\mathrm{Na}\right]}^{I}-\varepsilon {\left[A\right]}^{\mathrm{II}}{\left[\mathrm{Na}\right]}^{\mathrm{II}}\right)\right\}\\ D={\left[\mathrm{Na}\right]}^{I}{\left[\mathrm{Na}\right]}^{\mathrm{II}}\left(\varepsilon^{\prime }{\left[\mathrm{Tot}\right]}^{I}\left({\left[\mathrm{Tot}\right]}^{\mathrm{II}}+K_{\mathrm{ac}}\right)+\varepsilon {\left[\mathrm{Tot}\right]}^{\mathrm{II}}\left({\left[\mathrm{Tot}\right]}^{I}+K_{\mathrm{ac}}\right)\right)\\ \;+K_{\mathrm{ac}}K_{\mathrm{Na}}\left(\left(\varepsilon \prime +1\right){\left[\mathrm{Tot}\right]}^{I}{\left[\mathrm{Na}\right]}^{I}+\left(\varepsilon +1\right){\left[\mathrm{Tot}\right]}^{\mathrm{II}}{\left[\mathrm{Na}\right]}^{\mathrm{II}}\right)\\ \;+{K_{\mathrm{ac}}}^{2}K_{\mathrm{Na}}\left({\left[\mathrm{Na}\right]}^{I}+{\left[\mathrm{Na}\right]}^{\mathrm{II}}\right)+2{\left(K_{\mathrm{ac}}K_{\mathrm{Na}}\right)}^{2}\\ \varepsilon =e^{\left(\frac{\mathrm{\beta zF}}{\mathrm{RT}}\Delta \psi \right)}\;\mathrm{and}\;\varepsilon \prime =e^{\left(\frac{\left(\beta -1\right)\mathrm{zF}}{\mathrm{RT}}\Delta \psi \right)} \end{array}$$

where *[A]*^*i*^ and *[B]*^*i*^ are the concentrations (mol l^− 1^) of substrate *A* and *B* in compartment *i*, *[Tot]*^*i*^ is the sum of substrates *A* and *B*. *K*_*ac*_ and *K*_*Na*_ represent the dissociation constants (mol l^− 1^) of the amino acid substrates and sodium, respectively, and *V*_*ac*_ is the transport rate constant (mol min^− 1^). The electrical potential induced bias was given by *ε′* and *ε* for the forward and backward transport rate, respectively [26]. *β* represents the electrical bias constant, *z* is the charge of sodium, *F* is the Faraday constant, *R* is the gas constant, *T* is the absolute temperature and *∆ Ψ* is the membrane potential difference between side *I* and *II* (Table 2).

### 2.4 Compartmental blood flow

Blood flow in and out of the maternal and fetal compartments was modelled as constant (non-pulsatile). Compartments were assumed well mixed, with flow resulting in a net molecular flux (mol min^− 1^) as follows:

(7) $$J_{A,\mathrm{flow}}^{i}=F_{i}\left({\left[A\right]}_{\mathrm{in}}^{i}-{\left[A\right]}^{i}\right)$$

where *[A]*^*i*^_*in*_ is the input concentration (mol l^− 1^) of substrate *A* in compartment *i* and *[A]*^*i*^ is the concentration in the compartment. *F*_*i*_ is the flow rate in and out of compartment *i* (l min^− 1^).

### 2.5 Model parameters

Reference parameters are reported in Table 2. In general, the parameters selected were for normal physiological conditions. A single functional unit of the placenta (cotyledon) was modelled with a volume of 30 ml (~ 30 g), as described previously [20], [27]. For the transporter models, the transport rate constants *V* were initially taken equal for each class of transporter to clearly evaluate their influence on the system [20].

### 2.6 Numerical implementation

All models were implemented in Matlab (R2014a). To predict the concentrations of amino acids in each compartment, time integration of Eqs. (1), (3) was performed using the *ode45* function (Runge–Kutta (4, 5) method). Sensitivity analyses for the different model parameters were carried out based on steady state values of fetal amino acid transfer.

### 2.7 Parameter estimation

The effective transport rate parameters for each transporter included in the model (*V*_*ac*_*, V*_*ex* , *mvm*_*, V*_*ex* , *bm*_, and *V*_*fa*_) were fitted simultaneously based on the relative (normalised) error between the literature and predicted steady state fetal venous–arterial concentration difference. A least square criterion was used with all amino acid groups weighted equally. The fitting procedure was implemented using the *fminsearch* function in Matlab (Nelder–Mead method).

## 3 Results

This section will first explore how amino acids are transferred to the fetus across each syncytiotrophoblast plasma membrane (MVM and BM) separately. Subsequently, MVM and BM are combined, producing an integrated representation of how amino acids cross the placenta. Sensitivity analyses for model parameters are presented to understand the transport system as a whole and how these affect the different amino acid groups. Lastly, an example of the impact of a certain genetic condition with elevated phenylalanine levels (maternal phenylketonuria) is explored using the model.

### 3.1 Uptake of maternal amino acids: transport interactions across the microvillous plasma membrane

**Fig. 2:**
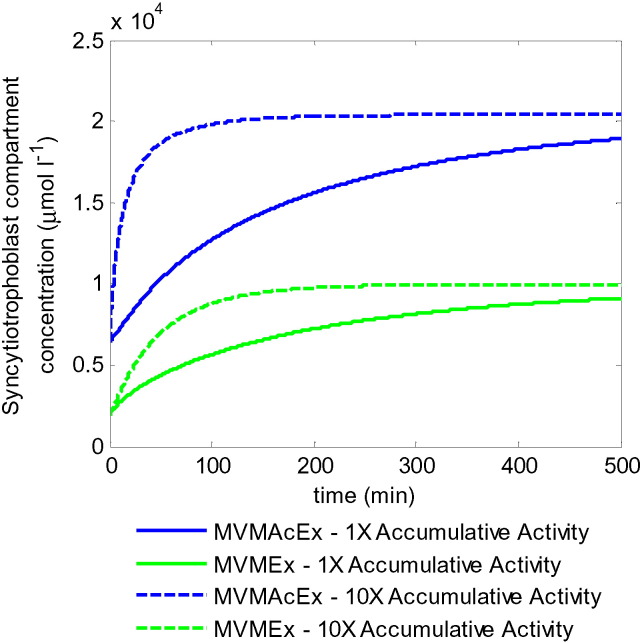
Model simulations of transport interactions at the MVM using physiological concentrations (Table 1). MVMEx consists of the sum of all amino acids transported by exchange only at the MVM, while MVMAcEx consists of the sum of those transported by exchange as well as accumulative transporters. Solid lines: equal accumulative and exchange transporter activities. Dashed lines: 10x increased accumulative transport activity. Note this does not affect equilibrium concentrations. Online version in colour.

Transport of amino acids across the MVM is mediated by both accumulative and exchange transporters (Fig. 1). While the accumulative transporters actively pump amino acids into the syncytiotrophoblast, the exchangers are responsible for equalising their relative composition. The amino acid substrates from Table 1 were categorised further into two groups according to their transporter specificity at the MVM: 1) Accumulative and exchange transporter substrate, MVMAcEx, consisting of AcEx and AcExF, and 2) Exchanger-only substrate, MVMEx, consisting of Ex and ExF. Physiological amino acid concentrations (Table 1) were combined and used as initial values for the maternal and syncytiotrophoblast compartments respectively and as constant input concentrations into the maternal compartment. Initially, transport across the BM was disabled to clearly demonstrate the potential for uptake across the MVM. The model showed concentrations in the syncytiotrophoblast rising well above maternal concentrations for both MVMAcEx and MVMEx (Fig. 2). This demonstrated that the combined accumulative and exchange transporter configuration allowed uptake of both amino acid groups across the MVM by transporting intracellular MVMAcEx substrates back out again from the syncytiotrophoblast in exchange for external MVMEx substrates. The syncytiotrophoblast concentrations of both substrate groups rose well above physiological values; however it is important to note that in this case the model only considered the MVM transporter activities and did not include efflux transport across the BM, which would lead to a lower equilibrium. Steady state concentrations were reached when the opposing gradients in accumulative transporter substrate and sodium electrochemical potential were equilibrated and the fraction of exchanger substrates equalised in both compartments. Thus, changes in transporter activity did not affect the equilibrium syncytiotrophoblast concentrations, but only the uptake rate and thus the speed at which this equilibrium was reached. This can be observed for the accumulative transporter activity in Fig. 2. In principle, higher exchanger activity could promote exchange of MVMAcEx back to the maternal side, leading to slower accumulation of MVMAcEx amino acids and faster uptake of MVMEx into the syncytiotrophoblast. However, increasing the exchanger activity by a factor 10 only had a minor impact, as the relative composition of both compartments already appeared to be in quasi steady equilibrium at any moment in time (results not shown).

### 3.2 Fetal delivery of amino acids: transport interactions across the basal plasma membrane

**Fig. 3:**
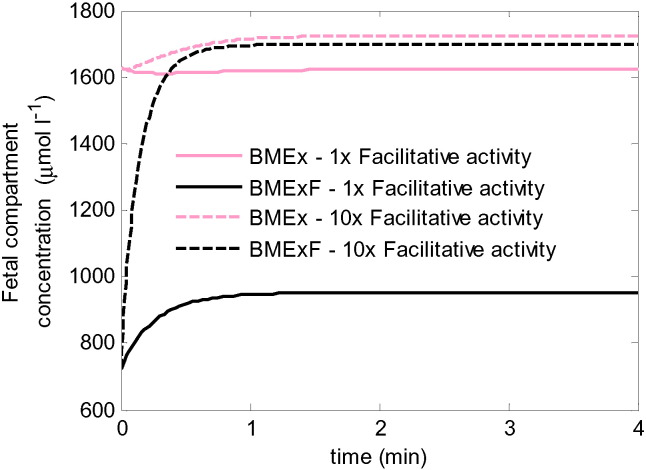
Model simulations of transport interactions at the BM using physiological concentrations (Table 1). BMEx is the sum of all substrates transported by exchange only at the BM, while BMExF is the sum of all substrates transported by both exchangers and facilitated transporters. Note that in the model the fetal compartment concentration equals that in the umbilical vein. Solid lines: equal exchanger and facilitative transporter activity. Dashed lines: 10x increased facilitative transport activity. For equal activity, the steady state level of BMEx at the end was slightly lower than the initial and input concentration, implying reverse transport from the fetal compartment into the syncytiotrophoblast.

Exchange and facilitative transporters localised to the BM are responsible for the delivery of amino acids to the fetus. While exchangers are important in regulating the relative composition of amino acids, it is the BM facilitative transporters which mediate net delivery of amino acids to the fetus. Thus, the interactions between these transporters were explored in Fig. 3. The amino acid concentrations from Table 1 were grouped further according to their transporter specificity at the BM: 1) Exchange substrates alone, BMEx, consisting of groups AcEx and Ex, and 2) Exchange and facilitative substrates, BMExF including ExF and AcExF. The combined umbilical arterial concentrations were used as both initial values and input concentrations for the fetal compartment, while in this case the syncytiotrophoblast amino acid concentrations were kept constant throughout the simulations. The results in Fig. 3 show an increase in fetal delivery for BMExF, evident from the increase in umbilical vein concentration over time. In contrast, a slight decrease in the fetal concentration of BMEx was observed, which implies reverse transport into the syncytiotrophoblast. This was due to the higher input fraction of BMEx in the fetal compartment (0.69) than the fraction in the syncytiotrophoblast (0.62), which led to reverse net transport due to exchange. However, it was shown that increasing the facilitative activity (e.g. by 10 fold) can indirectly boost the fetal delivery of BMEx to the fetus (Fig. 3). This is because the increased efflux of BMExF by the facilitative transporter reduced the fraction of BMEx in the fetal compartment, and once this fraction was lower than the fraction in the syncytiotrophoblast this then enabled net transfer to the fetus by the exchanger. However, lowering the fraction of BMEx in this way required a substantial increase in fetal compartment BMExF to a concentration much higher than physiological in the umbilical vein.

### 3.3 Transfer across the placenta from mother to fetus

**Fig. 4:**
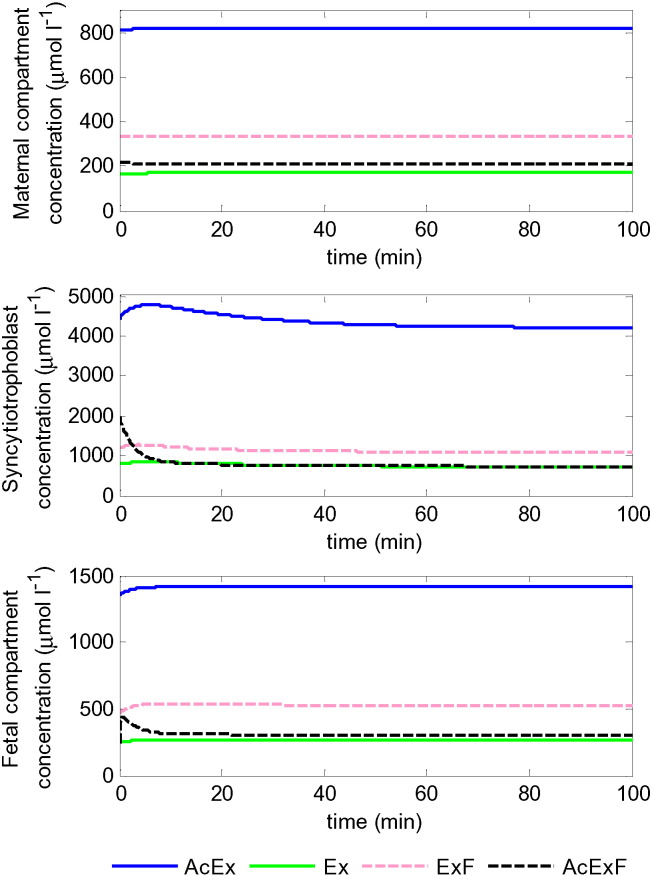
Model simulation of amino acid transfer across the placenta, showing the amino acid concentrations of the different groups of amino acids in each of the placental compartments. Simulations using physiological amino acid concentrations (Table 1) and model parameters from Table 2. Results represent the sum of all amino acids in each group. Note fetal AcExF rises sharply initially before going down again. Results demonstrated positive fetal delivery for all amino acids at steady state.

Having separately established the mechanisms of transport at the BM and MVM, the next step was to consider both membranes simultaneously. All three placental compartments were included (Fig. 1) and model simulations of the four groups of amino acids were generated using the physiological concentrations from Table 1 as inputs and initial conditions. The model simulations in Fig. 4 demonstrated that all four amino acid groups were successfully transferred to the fetal compartment, as evident from a net increase in their umbilical vein concentrations. Using literature values for maternal and fetal plasma as well as intracellular concentrations, the model appeared to be operating near steady state, although the amino acid groups AcEx and in particular AcExF showed reductions from the initial concentrations in the syncytiotrophoblast. Simulated results at steady state were compared with the umbilical venous–arterial concentration difference from literature [7] and appeared to correspond reasonably well on first inspection (Table 3), without any tuning of the model parameters. However, the model over-predicted transfer for amino acid groups AcExF and ExF to various degrees and under-predicted AcEx and Ex, with the greatest relative discrepancy being for Ex.

### 3.4 Effects of individual transporter activities

**Fig. 5:**
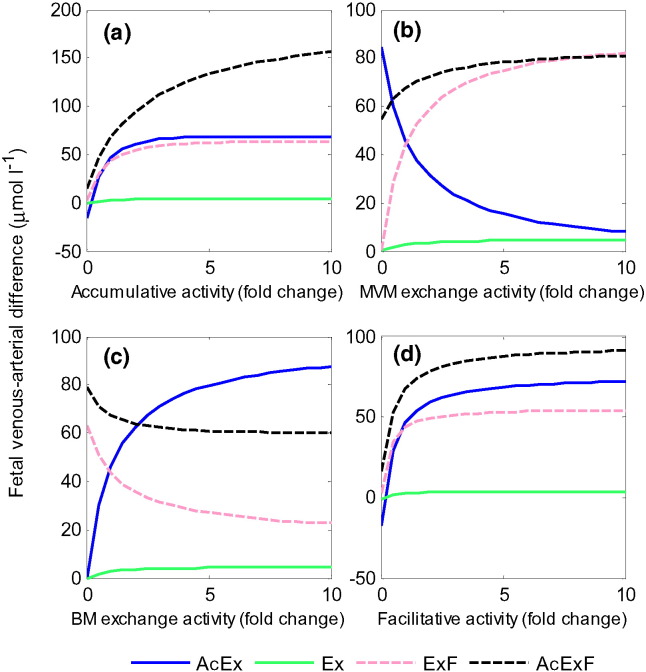
Individual transporter activity sensitivity analysis. Fetal venous–arterial difference at steady state for each amino acid group in response to variations in individual transporter activity with respect to the reference parameters (Table 2). Results represent the sum of all amino acids in each group.

The effect of varying the relative activity of each transporter type was explored. Reference transport activity parameters *V* for the accumulative, MVM exchange, BM exchange, and facilitative transporter (Table 2) were varied. Increasing the activities of accumulative and facilitative transporters promoted the placental transfer of all amino acid groups (Fig. 5a and d), until limits in placental transfer were reached. Interestingly, the results also showed that while increasing the activity of particular transporters promoted the transfer of certain amino acids, this was detrimental to the transfer of others. For example, increasing BM exchanger activity would result in a decrease in fetal delivery of amino acids that are transported by facilitative transporters (ExF and AcExF) (Fig. 5c), since this promotes exchange back into the syncytiotrophoblast. Similarly, increasing MVM exchanger activity promoted uptake and fetal delivery of those amino acids that are transported by exchange only at the MVM (Ex and ExF) at the expense of AcEx (Fig. 5b), which is taken up by the accumulative transporter and exchanged back into the maternal compartment. However, surprisingly an increase in placental transfer was observed for AcExF (Fig. 5b), which has the same accumulative-exchange transporter specificity at the MVM as AcEx. This is because in the reference simulation the syncytiotrophoblast fraction of AcExF dropped from a high initial ratio of 0.24 down to 0.11 at steady state, which is lower than the ratio of 0.14 on the maternal side. Increasing MVM exchange activity would then promote AcExF uptake into the syncytiotrophoblast compartment and in turn increase transfer to the fetal compartment by facilitated transport. Thus, MVM exchangers affected BM transfer indirectly, and in opposite manners depending on how the overall transport system shifted the concentration ratios of each amino acid in the three compartments. Lastly, it can be noted from Fig. 5a–d that the placental transfer of amino acid Ex (transported by exchanger only) could be driven by increasing any transporter activity, but only to a small degree. Negative fetal delivery, corresponding to amino acid transport out of the fetal compartment into the syncytiotrophoblast can occur for AcEx at very low facilitated (Fig. 5d) or accumulative (Fig. 5a) transporter activity.

### 3.5 Interactions between multiple transporter activities

**Fig. 6:**
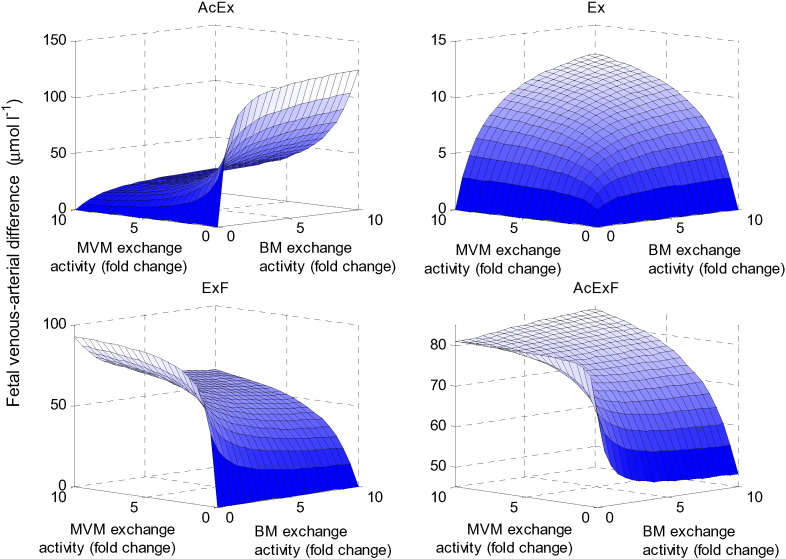
Effects of exchanger activity. Fetal venous–arterial difference at steady state for each amino acid group when the exchanger activities at the MVM and BM were varied simultaneously.

A series of simulations was performed in which two transporter activities were varied simultaneously to explore their interactions. First, the overall impact of exchanger activity on net placental transfer of each amino acid was explored by varying both MVM and BM exchanger activities (Fig. 6). This showed that for amino acid AcEx, increasing exchange activity at the BM while reducing exchange activity at the MVM would result in optimal fetal delivery (i.e. by promoting exchange to the fetus, while reducing back exchange to the maternal compartment). In contrast, for ExF and AcExF, both of which are facilitative substrates, increasing BM exchange activity could lead to reuptake into the syncytiotrophoblast. Interestingly, for AcExF, the BM exchanger activity had opposite effects on net transfer depending on whether the MVM exchanger activity was high or low. It was shown that in addition to having both exchanger activities high, additional high AcExF transfer could occur when both activities were low. This is because for low exchange activities the accumulative and facilitative transporters would dominate transfer, while back-exchange into the maternal and syncytiotrophoblast compartments is limited. For Ex, higher fetal uptake can be achieved by increasing both exchange activities, however, the overall transfer remained relatively small.

**Fig. 7:**
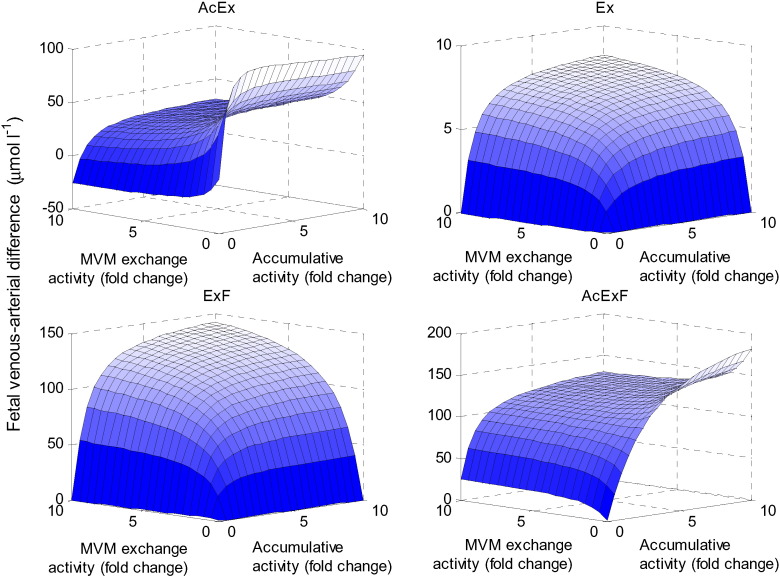
Effects of transporter activity at the MVM. Fetal venous–arterial difference at steady state for each amino acid group when varying the MVM exchanger and accumulative transporter activities simultaneously.

Next it was investigated how overall transport is affected by the transporters on the MVM, by simultaneously varying the accumulative and MVM exchange activities (Fig. 7). The results showed that maximum placental transfer of AcEx and AcExF occurred when the accumulative activity is high, which promotes uptake into the syncytiotrophoblast, and the exchange activity is low, which limits back-exchange. For Ex and ExF, the maximum delivery in the fetal compartment was achieved when both transporter activities at the MVM were high. This is because both transporters promote uptake via exchange into syncytiotrophoblast for these substrates, either directly or indirectly by increasing the intracellular concentrations of the driving substrates. Note that negative fetal delivery (transport out of the fetal compartment into the syncytiotrophoblast) occurred under certain conditions; for instance, for AcEx when the accumulative activity is low. This occurred because low MVM uptake of AcEx meant that its ratio in the syncytiotrophoblast was lower than on the fetal side, leading to reverse transport by BM exchange.

**Fig. 8:**
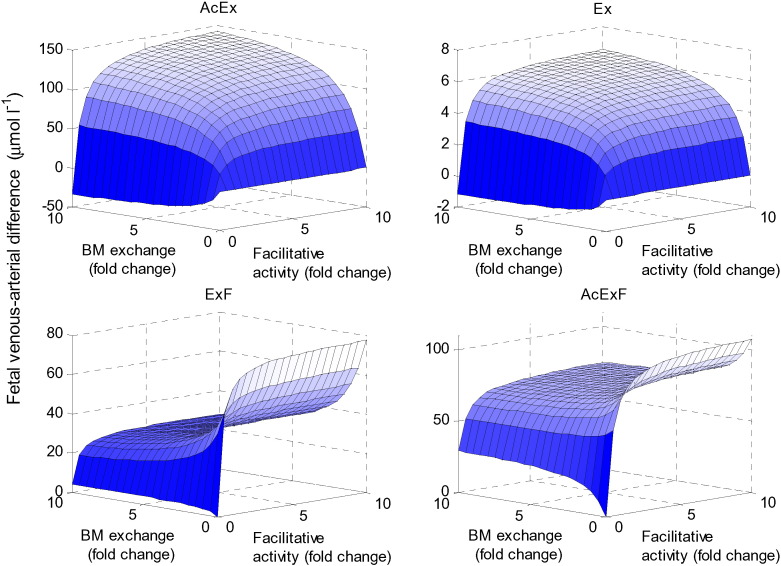
Effects of transporter activity at the BM. Fetal venous–arterial difference at steady state for each amino acid group when varying the BM exchanger and facilitative transporter activities simultaneously.

The impact of the transporter activities in the BM was evaluated by varying the activities of the BM exchanger and facilitative transporters (Fig. 8). The model suggested that for ExF and AcExF, the fetal delivery was optimal when the facilitative activity was high and the exchange activity at the BM was low. This combination promoted transfer to the fetus, while at the same time limiting reuptake. Additionally, it was shown that for AcEx and Ex, which are not substrates of the facilitative transporter, the fetal delivery was increased when all transport activities were high at the BM. These substrates must be exchanged to transfer across the BM, therefore promoting exchange will directly increase their transfer, and this is promoted indirectly by increasing the facilitative activity, since this leads to a more favourable exchange ratio.

### 3.6 Flow sensitivity

**Fig. 9:**
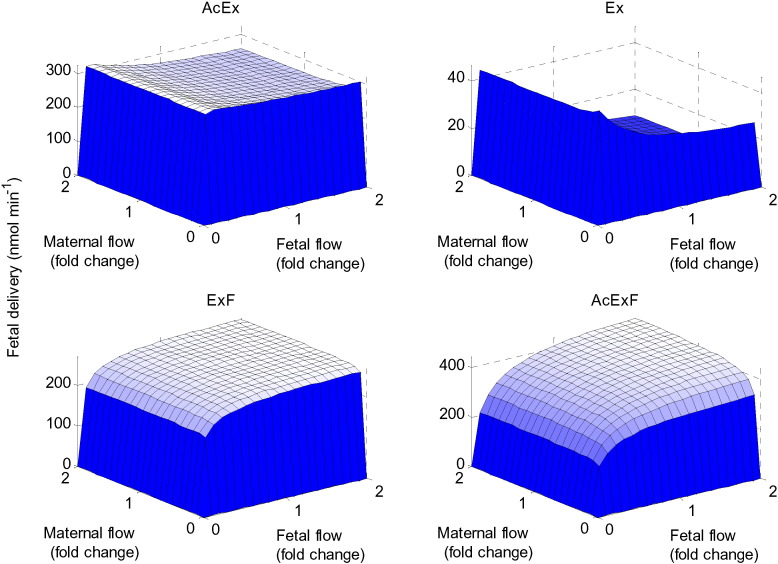
Flow sensitivity analysis. Net transfer to the fetus for each amino acid group in response to varying the maternal and fetal flow inputs simultaneously.

The impact of maternal and fetal blood flow on placental transfer was analysed for each amino acid group. Flow rates were only found to be rate limiting when either maternal or fetal flow approached zero. The system appeared to be most sensitive to changes in the fetal flow due to its small volume fraction (Fig. 9). In contrast, the maternal flow did not appear to significantly affect fetal delivery. The model suggested that a slow fetal flow rate translated to high fetal delivery for AcEx and Ex (not facilitative substrates), while faster fetal flow rate would stimulate fetal delivery of ExF and AcExF (facilitative substrates). This is because for the facilitative substrates, high fetal flow maintains the BM concentration gradients driving facilitated transport. While for amino acids that cannot be transported by facilitative transporters, high fetal flow would maintain the less favourable influx concentration ratios, which determine transport by the exchangers.

### 3.7 Model sensitivity to amino acid input concentrations

**Fig. 10:**
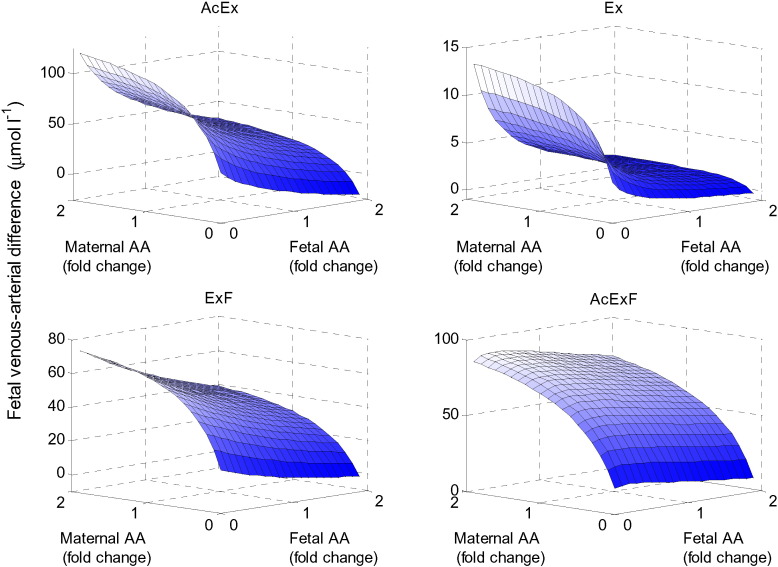
Effect of amino acid input concentrations. Fetal venous–arterial difference for each amino acid group in response to varying the overall maternal and fetal amino acid arterial input concentrations simultaneously. Note the concentrations of all amino acid groups were varied by the same factor.

The initial amino acid levels and input concentrations in the maternal and fetal compartments were varied using the same factor for all four amino acid groups with respect to their reference concentrations in Table 1. The results in Fig. 10 demonstrated that low fetal amino acid levels and high maternal amino acid levels generally promoted fetal transport, as would be expected. In addition, maternal amino acids became limiting only at low levels. The results also showed that negative net transfer could occur when maternal concentrations are extremely low and fetal concentrations are high.

### 3.8 Coordination of transporter activities

The model parameters were fitted to determine which combination of transporter activities would provide the best overall fit of the fetal venous–arterial concentration differences from literature [7] (Table 3). Compared to the reference simulations, fitting improved results especially for substrates that were initially under-predicted. AcEx was matched closely to the literature values (within 3.7%) and the low level of Ex was increased substantially. However, for AcExF and ExF the results deviated more from literature values than the original reference simulations. Compared to the reference parameters, the fitted transporter activities were adjusted by the following factors: accumulative × 0.75, exchange at MVM × 2.8, exchange at BM × 59, and facilitative activity × 1.3. This very large increase in BM exchanger activity reflects the attempt by the algorithm to match amino acid Ex and its low sensitivity.

### 3.9 Elevated maternal phenylalanine level

**Fig. 11:**
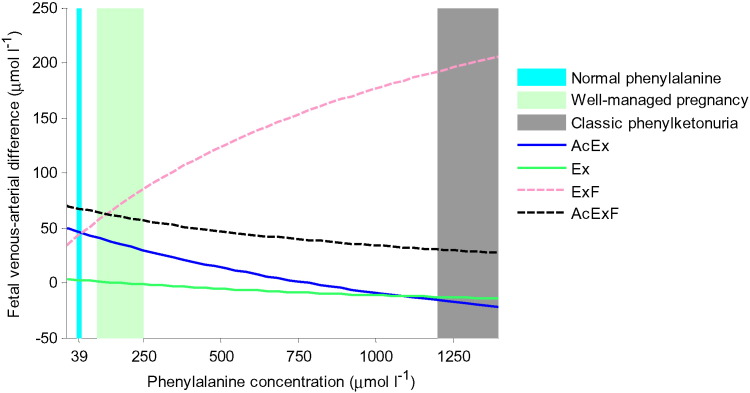
Effects of elevated maternal phenylalanine concentrations in phenylketonuria for each amino acid group. Note ExF rises as it includes phenylalanine, but all other amino acids are severely reduced. Normal phenylalanine levels are around 39 μmol l^− 1^, while for classic untreated phenylketonuria this exceeds 1200 μmol l^− 1^[37]. For well managed pregnancies where maternal phenylalanine is kept within the UK target range of 100–250 μmol l^− 1^ the model would still predict a significant reduction or even reversal in transfer of Ex, corresponding to threonine.

Finally, the model was used to explore the genetic condition of maternal phenylketonuria, where lack of phenylalanine hydroxylase causes an excess level of phenylalanine that can affect fetal development and function [37]. Phenylalanine is an exchange and facilitated transporter substrate. Therefore, net transfer of each amino acid group was modelled over a range of maternal ExF by including the additional phenylalanine. The results showed that elevated concentrations of ExF in the maternal compartment reduced the net transfer of all other amino acid groups (Fig. 11). Moreover, the model predicted negative net transfer of AcEx and Ex at high maternal phenylalanine concentrations, which implies that these amino acids were transported out of the fetal compartment.

## 4 Discussion

This study developed an integrated modelling approach to explain the interaction of transporters polarised to the microvillous apical and basal plasma membranes of the human placental syncytiotrophoblast. The modelling framework developed was effectively able to represent the complexity arising from the interactions between multiple species of amino acids and different types of transporters on both the MVM and BM. This will prove invaluable in determining the contribution of specific transporters to epithelial transport in the placenta and other transport systems. The ability to predict how specific transporters contribute to overall function will allow the design of targeted interventions in epithelial transport disorders.

The model first successfully described the fundamental transporter interactions at each of the placental plasma membranes separately, before these were combined for the system as a whole. The accumulative-exchange transporter configuration at the MVM allowed the accumulation of all the different types of amino acids into the syncytiotrophoblast. Indirect stimulation of amino acids that were not substrates of the accumulative transporter could be achieved by increasing the accumulative transporter activity to promote exchange. The syncytiotrophoblast uptake concentrations of both accumulative and exchange amino acid species were substantially higher than the maternal concentrations. This accumulation against the concentration gradient is enabled by the energy required to maintain the constant sodium gradient whose electrochemical potential provides the driving force for the system. Similarly, the model confirmed that the facilitative-exchange transporter configuration at the BM was sufficient to ultimately transfer all amino acids to the fetus. In addition, indirect stimulation of amino acids that were not a substrate of the facilitative transporter was shown to be possible by increasing the facilitated transport activity to promote exchange across the BM.

When the overall transfer across the placenta was considered using physiological concentrations, the integrated model operated close to steady state (Fig. 4) and showed a favourable net transfer of all amino acid groups to the fetus (Table 3), in reasonable agreement with literature [7]. This indicated that the model could provide a relatively robust representation of placental amino acid transfer, despite many simplifying assumptions. Fitting results suggested that the model predictions could be improved by changing the activities for each transporter. Though, it appeared difficult to adjust independently the concentration of certain amino acid groups without affecting the transfer of others. In particular, improving the prediction for the exchange only substrate required a disproportional increase in BM exchanger activity (Table 3).

Simultaneous variation of the transporter activities revealed that multiple configurations could result in high transfer for certain amino acids (AcExF in Fig. 6). Amino acids groups that were substrates of the accumulative transporter (AcEx and AcExF) generally behaved in the same way when considered at the MVM, in contrast with those that were not accumulative transporter substrates (Ex and ExF, Fig. 7). Similarly, amino acid groups that were substrates of the facilitative transporter (ExF and AcExF) displayed the same response when observed at the BM, showing a distinctly different response compared with those that were not transported by the facilitative transporter (AcEx and Ex, Fig. 8). Against a background where strategies are being developed to specifically target placenta to deliver pharmacological or genetic therapies [38], modelling may allow more informed decisions as to which transporters to target. However, the differential effect on different amino acids by changing transporter activity should serve as a cautionary warning that potential unwanted side effects may be elicited by an intervention.

Simulation results were shown to be most sensitive to fetal, rather than maternal flow due to the low compartmental volume. However, compared to the physiological reference values, increasing flow further did not lead to significant changes in transfer (Fig. 9). Flow rates were only shown to be rate limiting when either maternal or fetal flow rates approached zero. Under the given conditions, substrates of the facilitative transporter displayed a positive relationship with flow, whereas the other amino acids showed the opposite behaviour. Maternal uterine and fetal umbilical blood flows are one of the few placental parameters that can currently be measured in vivo and this model may provide a basis for understanding their effect on the fetus.

It has to be emphasized that the current model was for the placenta and did not include maternal or fetal metabolism, which will alter amino acid availability in the maternal or fetal arteries. However, as demonstrated by the model, when overall maternal and fetal amino acid concentrations were varied, it was shown that placental transfer was increased by both high maternal and low fetal concentrations of amino acids, as would be expected. Nonetheless, placental transfer was mainly controlled by fetal concentrations, suggesting that amino acid transfer to the fetus is in part regulated by fetal demand. Furthermore, this means that increasing overall maternal amino acid concentrations above physiological levels is unlikely to be an effective intervention strategy. However, interventions could also specifically target changing the relative amino acid composition which is important for exchanger function, as informed by the model. This is particularly relevant for inherited diseases of metabolism that affect the concentration of specific amino acids as shown by the model example of phenylketonuria, where maternal phenylalanine levels can be over 30 × higher than normal [37], severely impairing placental transfer of other amino acids (Fig. 11).

It is important to consider the simplifying assumptions made in deriving the current model. In particular, amino acids were grouped according to their specificity for the various transporter types and each transporter type was modelled as a single representative transporter. In reality, numerous different individual transporters can be distinguished for each transporter type, each of which is specific to certain overlapping subsets of amino acids, with different substrate affinities [12]. In addition, since the main focus was on the function of the transporters as a coordinated system, individual transporter models were kept relatively simple, in order to capture faithfully the underlying mechanisms for each type, while minimising the number of unknown parameters. For example, translocation and binding were assumed symmetric for the exchanger and facilitative transporter, however this is not an intrinsic limitation and given enough available data this assumption can be relaxed within the thermodynamic constraints for an energetically passive transport process [21], [23]. For the active accumulative transporter the ultimate level of amino acid accumulation is determined by the sodium electrochemical potential difference, independent of the kinetics. The effect of membrane potential was included for sodium in the accumulative transporter, however an important limitation of the current model was that Eqs. (4), (6)only describe the transport of neutral amino acids. Therefore the amino acid groups considered in the present model should be subdivided further to specifically model the transport of charged amino acids by transporters such as system y^+^. In addition, placental metabolism was not considered in the model, as the main focus was on transport; this could potentially change the amount of amino acids available for transport and their relative composition. Another aspect not included in the model was transfer via paracellular routes, which are poorly understood anatomically [39]. Paracellular diffusion will reduce the efficiency of the system because of high fetal amino acid concentrations, causing net diffusion in the fetal to maternal direction. All compartments in the model were assumed well-mixed, ignoring differences in local concentrations due to the maternal intervillous and fetal capillary flow. In addition, this implied that the intracellular concentrations in the syncytiotrophoblast were assumed uniform, rather than forming a gradient. Including diffusion within the syncytiotrophoblast in the model did not affect results, as the estimated timescale for diffusion is fast compared to membrane transport in our model (*T* = *h*^*2*^*/D* *=* 0.1 s, with thickness *h* < 1 × 10^− 5^ m and diffusion coefficient *D* = 1 × 10^− 9^ m^2^/s [40]). Nonetheless, further compartmentalisation within the syncytiotrophoblast could be important and this could lead to differences in the intracellular concentrations determining transport at the BM and MVM.

While the model was designed for amino acids, the transporters included in the model also transfer a wide range of other substances including xenobiotics. As such, the modelling framework can be widely applicable to transport functions both in the placenta and other transporting epithelia; for instance intestinal absorption of nutrients and drugs, reabsorption of nutrients from the renal tubules, and the transfer of nutrients and drugs across the blood brain barrier [41].

In summary, a novel integrated modelling framework was developed for the placental amino acid transfer system as a whole. The model was shown to be able to capture successfully the principal features of the transfer system despite the necessary simplifying assumptions. Transporter modelling is currently limited by the availability of specific details about individual transporters, their kinetics and substrate specificity. However, one of the strengths of this modelling framework is that it can easily be updated as experimental data becomes available. To illustrate the potential of the model for representing clinical scenarios, the case of phenylketonuria was modelled; demonstrating how elevated maternal phenylalanine would restrict fetal delivery of all other amino acids. Ultimately it is hoped that this type of modelling approaches will inform biological understanding and aid the development of targeted intervention strategies.

## Funding

RL, CP and BS were funded by BBSRC grant BB/I011315/1 and KW, IC, EJ, CS and JG by BB/I011250/1. NP received EPSRC DTP funding.

## Data availability statement

Matlab source code available at http://dx.doi.org/10.5258/SOTON/385034.

## Conflict of interest

We declare we have no conflicts of interest.

## Transparency document

Transparency document

## Figures and Tables

**Table 1:** Amino acids groups distinguished in the model as categorised by transporter specificity and their physiological concentrations [7], [8]^a^.

Amino acid group	Transporter specificity	Name	Maternal concentration (μmol l^− 1^) [7]	Syncytiotrophoblast concentration (μmol l^− 1^) [8]	Umbilical artery concentration (μmol l^− 1^) [7]	Umbilical vein concentration (μmol l^− 1^) [7]
AcEx	Accumulative and exchange	Arginine	51	320	101	108
		Glutamine	368	1135	482	494
		Glycine	107	1655	217	218
		Histidine	85	74	108	119
		Lysine	131	508	317	343
		Serine	84	721	144	142
		**Sum**	**826**	**4413**	**1369**	**1424**
Ex	Exchange	Threonine	**170**	**786**	**258**	**270**
ExF	Exchange and facilitative	Isoleucine	43	105	56	63
		Leucine	81	305	105	120
		Phenylalanine	39	173	63	65
		Tyrosine	34	220	59	59
		Valine	141	342	200	216
		**Sum**	**338**	**1145**	**483**	**523**
AcExF	Accumulative, exchange, and facilitative	Alanine	**215**	**1987**	**238**	**282**

Values indicated in bold represent the total concentrations for each amino acid group used in the model.

a Note, glutamate and aspartate were excluded as these are taken up by distinct transport systems. Asparagine, proline and methionine were not included as the data sets used did not contain values for all three compartments.

**Table 2:** Reference parameter values.

Parameter	Value	Unit	Reference
*Compartmental model:*			
Total placental cotyledon volume	30	ml	[10], [20]
% maternal volume (intervillous space)	34%	–	[20], [27]
% syncytiotrophoblast volume	15%	–	[20], [27]
% fetal volume (fetal capillaries)	7.5%	–	[20], [27]
Maternal blood flow rate	2	ml min^− 1^ g^− 1^	[28]
Fetal blood flow rate	0.2	ml min^− 1^ g^− 1^	[29]

*Transporter models:*			
Accumulative			
Effective transport rate, V_ac_	0.005	mmol min^− 1^	[20]
Charge of sodium ion, z	1	–	–
Faraday constant, F	9.65 × 10^4^	C mol^− 1^	–
Gas constant, R	8.314	VC K^− 1^ mol^− 1^	–
Body temperature, T	310	K	–
Transmembrane potential difference, ∆ Ψ	− 21	mV	[30]
Maternal sodium concentration	134	mmol l^− 1^	[31]^a^
Syncytiotrophoblast sodium concentration	15	mmol l^− 1^	[32]^b^
Electrical bias constant, β	0.33	–	[33]^c^
Substrate dissociation constant, K_ac_	2.26	mmol l^− 1^	[33]^c^
Sodium dissociation constant, K_Na_	25.07	mmol l^− 1^	[33]^c^
MVM exchanger			
Maximum transport rate, V_ex,mvm_	0.005	mmol min^− 1^	[20]
Substrate dissociation constant, K_ex_	200	μmol l^− 1^	[34], [35]^d^
BM exchanger			
Maximum transport rate, V_ex,bm_	0.005	mmol min^− 1^	[20]
Substrate dissociation constant, K_ex_	200	μmol l^− 1^	[34], [35]^d^
Facilitative			
Maximum transport rate, V_f_	0.005	mmol min^− 1^	[20]
Substrate dissociation constant, K_f_	1000	μmol l^− 1^	[36]^d^

a Mean of lower and upper late pregnancy values.

b Intracellular value for brain as no specific information available.

c Fitted based on data Yao et al., Fig. 5f [24], [33].

d Representative value based on weighted average dissociation constants.

**Table 3:** Umbilical venous–arterial concentration differences for each amino acid group.

Amino acid group	Reference simulation (μmol l^− 1^)	Literature [7] (μmol l^− 1^)	Model fit (μmol l^− 1^)
AcEx	46	55	53
Ex	2.7	12	9.3
ExF	43.6	40	32.5
AcExF	67.4	44	70
